# Adherence and barriers to penile rehabilitation over 2 years following radical prostatectomy

**DOI:** 10.1186/s12894-019-0516-y

**Published:** 2019-10-07

**Authors:** Jeffrey Albaugh, Brittany Adamic, Cecilia Chang, Nicholas Kirwen, Joshua Aizen

**Affiliations:** 1 Northshore University HealthSystem, Evanston, IL USA; 2 University of Chicago Medical Center, Chicago, USA

**Keywords:** Penile rehabilitation, Radical prostatectomy, Erectile dysfunction, Adherence

## Abstract

**Background:**

A variety of penile rehabilitation (PR) therapies are available to improve post-prostatectomy erectile dysfunction (ED) with mixed results. It is uncertain how adherent men are to PR therapies. The aim of this study is to determine adherence to and identify barriers to PR treatment.

**Methods:**

A longitudinal cross-sectional approach was used in men who underwent radical prostatectomy over 2 years. Men were instructed to take a PDE5 inhibitor (PDE5i) three times per week, and if required, utilize a vacuum constriction device (VCD) daily. Outcomes were measured by multiple validated questionnaires. In addition, penile stretched length, side effects, compliance to PR regimen & barriers to participation were documented.

**Results:**

Seventy-seven patients were enrolled, however only 49 completed evaluation at 3 or more timepoints and were included in analysis. This cohort was an average age of 58.1 years (±7.7), had robotic laparoscopic radical prostatectomy (91.7%), and had bilateral nerve sparing procedures (95.8%). Majority (62.5%) reported normal SHIM pre-operatively, however 79% used PDE5i. Erectile function as measured by IIEF and Erection Hardness Rating were negatively affected post-operatively, with gradual improvement in parameters throughout the 24 month follow up. Of the participants who had normal pre-op SHIM, only 23.1 and 28.6% regained baseline function at 1 and 2 years, respectively. Orgasm was significantly diminished immediately post-operatively, however, at the end of the study period only 37% of men reported diminished climax and no men reported absent orgasm. Adherence to penile rehabilitation therapies declined overtime. Men took oral PDE5i on average 2.3 times weekly at 12 and 24 months (*p* < 0.001). Men used the VCD 2.3–3.9 days a week, which declined overtime (*p* = 0.014).

**Conclusions:**

Improvement in erectile and orgasm parameters was observed over time, but most men did not return to baseline function. Despite comprehensive instructions and a frequent follow up schedule, PDE5i and VCD adherence was poor. High attrition rates were noted with only 55.8% of men remaining at 12 months and 45% of men completing 24 months. The most common barriers to PR adherence were cost, inconvenience and perceived ineffectiveness.

## Background

Prostate cancer is the most commonly diagnosed non-skin cancer in men [1]. Men with clinically significant prostate cancer are required to decide between treatment options, each with side effects that may greatly affect quality of life [2]. Despite advances in nerve-sparing surgical techniques, the incidence of erectile dysfunction 2–15 years following radical prostatectomy has been reported to be as high as 78–87% in a large population-based study [3]. Erectile recovery following nerve-sparing radical prostatectomy can take two or more years [4].

The goal of penile rehabilitation (PR) after prostate cancer treatment is to maximally preserve preoperative erectile function through the use of medications and/or devices. PR success is determined by the ability to achieve and maintain erectile rigidity sufficient for satisfactory sexual relations without the use of any further ED treatments then used prior to surgery. Treatment options for PR include oral phosphodiesterase type 5 inhibitors (PDE5i), vacuum constriction devices (VCD), and intracavernosal injections (ICI). Men with comorbid medical conditions or those who utilized medications for erectile dysfunction prior to prostate cancer treatment are at greater risk for erectile dysfunction following radical prostatectomy [5].

Recommendations from the fourth International Consultation for Sexual Medicine suggest discussing erectile dysfunction and PR options with men prior to and after radical prostatectomy. The panel determined that inadequate data exist to recommend any specific PR regimen over another [6]. Thus, a critical need exists for further research in the realm of post-prostatectomy PR to determine the most effective strategies. This study aimed to assess rates of PR adherence and identify barriers to participation in PR. It also sought to assess the progression of erectile function and orgasm quality in men utilizing a post-prostatectomy PR regimen.

## Methods

The study utilized a longitudinal cross-sectional approach in which men who underwent radical prostatectomy were followed for 2 years following surgery with assessment at 1, 3, 6, 12 and 24 month intervals after beginning penile rehabilitation. The Sexual Health Inventory for Men (SHIM) [7], a five-item subscale within the International Index of Erectile Function (IIEF) that assesses presence and severity of erectile dysfunction, was completed by all patients preoperatively [8]. Men underwent radical prostatectomy via a nerve-sparing approach when feasible from a technical and oncologic standpoint. Post-operatively, men initiated a PR regimen, consisting of PDE5i with or without VCD and/or ICI. Specifically, participants were instructed to take sildenafil with varied doses up to 100 mg or tadalafil up to 20 mg as needed at least three times per week. Participants could take doses of sildenafil or tadalafil, with dosing as needed as tolerated. Men were encouraged to take a dose a minimum of 3 times a week and did not take daily doses of tadalafil 5 mg. The men were encouraged to take varied doses three times a week as needed as tolerated rather than daily to determine if any doses were helping as compared to no medication. After taking sildenafil or tadalafil, patients were asked to complete an Erection Hardness Score (EHS), a scale ranging from 0 (no erection) to 4 (fully hard erection) [9]. If they were unable to achieve ≥3 on this scale with oral agents alone, they were instructed to add VCD to their regimen. Prescribed VCD routines consisted of 5–10 stretches per day, each held for 1–2 min within the device. If patients desired, they were also instructed to use intracavernosal injections as an adjunct erectogenic therapy.

Men were included in this study if they had undergone radical prostatectomy ≤12 weeks before initiating PR. Only men with preoperative SHIM of ≥16 were included, confirming at least moderate erectile function at baseline. Patients were required to have a sexual partner at the time of treatment. Finally, participants were excluded if they were unable to safely be treated with PDE5i and/or VCD. Informed consent was obtained from all participants for this institutional review board approved study.

Prior to the study, baseline preoperative clinical parameters were recorded from the medical record when available or from the patient, including co-morbidities, baseline erectile function and orgasm quality, and stretched penile length. During the course of the study, a variety of self-reported measures were completed by participants at the following time points: immediately post-surgery, 1 month, 3 months, 6 months, 12 months, and 24 months post-operatively. Some of the measures included the Erectile Dysfunction Inventory of Treatment Satisfaction (EDITS) [10], EHS, IIEF (15-item questionnaire measuring erectile function, orgasmic function, sexual desire, intercourse satisfaction, and overall sexual satisfaction) including the Sexual Health Inventory for Men (SHIM/IIEF-5), Self-Esteem and Relationship Questionnaire (14-item patient-reported assessment of sexual confidence and intimacy) [11], and stretched penile length. A list of the data collected/instruments/measures are described in the Appendix. The SHIM scores are collected with and without treatment. With treatment (treated SHIM) score means the men were responding to SHIM questions with the use of their erectile dysfunction treatment if needed (oral agents, vacuum device and/or injections) and without treatment (untreated SHIM) means they were not using anything to treat their erectile dysfunction (unless they were taking oral meds before surgery and then they would only be using what they used prior to surgery with the untreated score). In addition to these instruments, clinical data, PR side effects, compliance to PR regimen, and barriers to participation were documented at each follow up visit. Although it was not the main aim of the study, we also measured quality of life with the Quality of Life Index-Cancer Version (QLI- a validated measurement of overall quality of life, health and functioning, social and economic functioning, psychological/spiritual stability, and family role) [12] and the UCLA Prostate Cancer Index (measurement of health-related quality of life in men treated for prostate cancer) [13]. Categorical variables were analyzed using chi-squared test, whereas continuous variables were compared over time using ANOVA. All statistical analyses were conducted in SAS 9.4 (SAS Institute, Cary, NC).

## Results

Seventy-seven men were enrolled in the study, however only 49 men were included in final analysis, as those who completed less than 3 evaluations were excluded. Table 1 describes the demographics of our cohort. The average age of our participants was 58.1 (SD = 7.74) and majority of men were Caucasian (89.8%). This cohort was well educated with 89.6% having a college level education or greater. Nearly all men underwent robotic nerve sparring radical prostatectomy (91.7%) with majority undergoing bilateral nerve sparring (95.8% bilateral and 4.2% unilateral). Seventy-nine percent of men reported treatment for erectile dysfunction (ED) pre-operatively with a PDE-5 inhibitor. The majority had a normal SHIM (62.5%) with a mean SHIM of 22.2 (SD = 2.59) and a mean erection hardness score of 3.41 out of 4 (SD = 0.37). Average stretched penile length was 13.7 cm (SD = 1.83) prior to prostatectomy.

**Table 1:** Initial Demographics

	Baseline (*N* = 49)	
	N	%
Age	58.12 ± 7.74	
< 50	8	16.33
50–59	20	40.82
60–69	17	34.69
≥ 70	4	8.16
Race		
Caucasian	44	89.80
African-American	5	10.20
Highest Education Level		
High School	5	10.42
Some College	6	12.50
Associate Degree	1	2.08
Bachelor’s Degree	13	27.08
Master’s Degree	15	31.25
Doctorate Degree	8	16.67
Type of Surgery		
Robotic	44	91.67
Open	4	8.33
Nerve Sparring		
Nerve Sparring	46	95.83
Non-nerve Sparring	0	0.00
Unilateral or Partial Nerve Sparring	2	4.17
ED Medications		
No	11	22.45
Yes	38	77.55
Any Treatment for ED		
No	10	20.83
Yes	38	79.17
Pre-Op SHIM	22.20 ± 2.59	
Abnormal	18	37.50
Normal (22–25)	30	62.50
Post-Op SHIM	4.78 ± 4.07	
Abnormal	49	100.00
Normal (22–25)	0	0.00

Post-operative functional evaluations can be found in Tables 2 and 3. The treated SHIM at 3 months post-operatively was 12.13 (± 7.57) with only 21.7% of men reporting a normal erection. The SHIM and IIEF scores did improve over the 2 year follow up, with 40% of men reporting a normal treated SHIM at 2 years. Men without ED pre-operatively, as defined by a SHIM of 22–25, did not adequately return to baseline erectile function. Only 23.1 and 28.6% of these men reported a normal treated SHIM at 1 and 2 years respectively without ED treatment. Despite poor erectile function as measured by SHIM, mean treated erection hardness ratings returned to baseline at 2 years when using erectile dysfunction treatments. On average, loss of penile stretched length was 1.76 cm at the one-month post-operative visit. Average penile stretched length returned to within 0.5 cm of baseline after 6 months of penile rehabilitation and was maintained throughout the remainder of the follow up period. Overall, quality of life was not significantly impacted as measured by the Quality of Life Index, however, the health and functioning domain improved over the 2 year study period from 23.7 ± 4.10 to 26.1 ± 2.96 out of 30 (*p* = 0.049). The overall SEAR scores, measuring relationship satisfaction and sexual self-esteem were low following surgery but improved significantly over time as measures of erectile function improved.

**Table 2:** Patient-Reported Outcomes of Erectile Function and Relationship Satisfaction over Time

	Pre-Op (*N* = 49)	Baseline (*N* = 49)		1 Month (*N* = 48)		3 Months (*N* = 46)		6 Months (*N* = 45)		1 Year (*N* = 43)		2 Years (*N* = 35)		*p*-value^b^
Treated SHIM Score^a^	22.20 ± 2.59	4.78 ± 4.07		9.88 ± 6.96		12.13 ± 7.57		14.80 ± 7.71		14.23 ± 8.80		16.66 ± 7.78		<.0001
Untreated SHIM Score				6.46 ± 5.04		7.70 ± 5.71		9.91 ± 6.32		12.55 ± 6.88		15.31 ± 6.92		<.0001
EDITS Score^e^				63.80 ± 18.83		62.04 ± 19.54		67.33 ± 22.35		71.75 ± 22.74		78.40 ± 20.93		0.0042
IIEF Score														
Erectile Function (EF)		5.80 ± 4.61		12.38 ± 8.35		15.20 ± 9.13		18.02 ± 9.52		17.51 ± 10.80		20.54 ± 9.48		<.0001
Orgasmic Function (OF)		4.10 ± 3.66		6.71 ± 2.68		6.93 ± 2.71		7.49 ± 2.42		6.63 ± 3.38		7.77 ± 2.45		<.0001
Climax/Orgasm														
Absent		8	18.60	2	4.26	0	0.00	3	6.67	1	2.33	0	0.00	0.0029
Diminished		20	46.51	21	44.68	17	36.96	15	33.33	11	25.58	13	37.14	
Normal		14	32.56	9	40.43	21	45.65	18	40.00	25	58.14	12	34.29	
Better		1	2.33	5	10.64	8	17.39	9	20.00	6	13.95	10	28.57	
Sexual Desire (SD)		6.43 ± 2.27		7.21 ± 1.57		7.02 ± 2.02		7.20 ± 1.74		6.65 ± 2.74		7.09 ± 1.99		0.3615
Intercourse Satisfaction (IS)		3.10 ± 3.20		5.77 ± 4.42		6.72 ± 4.51		8.16 ± 3.81		7.95 ± 4.65		8.57 ± 4.07		<.0001
Overall Satisfaction (OS)		4.90 ± 2.26		6.10 ± 2.41		6.09 ± 2.34		6.42 ± 2.49		6.16 ± 3.20		6.94 ± 2.61		0.0105
Treated EHR^c^	3.41 ± 0.37	1.15 ± 0.87		1.82 ± 1.02		2.56 ± 0.98		2.89 ± 0.85		3.21 ± 0.79		3.54 ± 0.42		<.0001
Untreated EHR				1.41 ± 0.87		1.68 ± 0.92		2.02 ± 1.06		2.55 ± 1.07		3.10 ± 0.70		<.0001
Penile Stretched Length	13.73 ± 1.83	11.97 ± 1.63		12.34 ± 1.48		12.98 ± 1.45		13.37 ± 1.38		13.42 ± 1.32		13.80 ± 1.24		<.0001
SEAR^d^		17.47 ± 8.75		20.60 ± 7.70		22.22 ± 8.49		24.91 ± 9.11		26.79 ± 8.62		32.34 ± 10.94		<.0001
Sexual Relationship Satisfaction		71.56 ± 19.77		70.05 ± 24.80		73.10 ± 24.11		80.69 ± 15.76		80.67 ± 15.30		84.82 ± 15.99		0.0021
Self-Esteem		78.06 ± 20.97		76.82 ± 20.14		78.80 ± 25.80		80.83 ± 17.80		81.10 ± 20.30		82.50 ± 20.83		0.8233
Overall Relationship Satisfaction		48.51 ± 18.79		53.50 ± 19.22		57.53 ± 21.51		64.80 ± 20.10		68.19 ± 18.60		74.49 ± 19.71		<.0001
Total		17.47 ± 8.75		20.60 ± 7.70		22.22 ± 8.49		24.91 ± 9.11		26.79 ± 8.62		32.34 ± 10.94		<.0001

^a^Data are presented as mean ± SD or N and %; ^b^ANOVA for *p*-values over time from baseline to 2 years; ^c^*EHR* Erection Hardness Rating; ^d^*SEAR* Self Esteem and Relationship; ^e^*EDITS* Erectile Dysfunction Inventory of Treatment Satisfaction

**Table 3:** SHIM Scores over Time

	1 Month (*N* = 48)		3 Months (*N* = 46)		6 Months (*N* = 45)		1 Year (*N* = 43)		2 Years (*N* = 35)		*p*-value
	N	%	N	%	N	%	N	%	N	%	
Treated SHIM Score^a^	9.88 ± 6.96		12.13 ± 7.57		14.80 ± 7.71		14.23 ± 8.80		16.66 ± 7.78		0.0010
Abnormal	42	87.50	36	78.26	31	68.89	30	69.77	21	60.00	0.0500
Normal (22–25)	6	12.50	10	21.74	14	31.11	13	30.23	14	40.00	
Untreated SHIM Score^a^	6.46 ± 5.04		7.70 ± 5.71		9.91 ± 6.32		12.55 ± 6.88		15.31 ± 6.92		<.0001
Abnormal	47	97.92	43	93.48	43	95.56	36	83.72	28	80.00	0.0176
Normal (22–25)	1	2.08	3	6.52	2	4.44	7	16.28	7	20.00	
SHIM scores similar to initial					Normal Pre-Op SHIM scores (*N* = 30)						
	1 Year (*N* = 43)		2 Years (*N* = 35)				1 Year (*N* = 26)			2 Years (*N* = 21)	
	N	%	N	%			N		%	N	%
Treated SHIM Score					Treated SHIM Score						
Dissimilar	36	83.72	30	85.71	Abnormal		18		69.23	14	66.67
Similar	7	16.28	5	14.29	Normal		8		30.77	7	33.33
Untreated SHIM Score					Untreated SHIM Score						
Dissimilar	40	93.02	30	85.71	Abnormal		20		76.92	15	71.43
Similar	3	6.98	6	14.29	Normal		6		23.08	6	28.57

^a^Data are presented as mean ± SD or N and %; Normal SHIM Score = (22–25)

Orgasm was also impacted by prostate cancer treatment. The orgasm function domain of the IIEF (Questions 9–10 with maximum score of 10) was significantly diminished post-operatively (4.1 ± 3.66), however improved with a mean score of 7.77 ± 2.45 at 24 months. At the end of the study period, only 37% of men reported diminished climax, with no men reporting absent orgasm.

Satisfaction with treatment, as measured by the EDITS improved significantly over time from an average of 63.8 (SD = 18.8) at 1 month to 78.4 (SD = 20.9) at 24 months (*p* = 0.004). Adherence to the penile rehabilitation protocol declined with time after prostatectomy as seen in Table 4. Men were adherent to oral PDE5i, using them 3 times per week initially post-op, which declined to 2.3 times per week on average at 24 months. Men instructed to utilize a vacuum device daily reported use on average 4.5 days per week at 1 month post-operatively, declining to 3.12 days per week at 24 months. Only 2–5% of men utilized the VCD five or more times per week. The utilization of a VCD was highest between months 3–12 (60.5–71.7%).

**Table 4:** Treatment Adherence and Compliance over Time

	1 Month (*N* = 48)		3 Months (*N* = 46)		6 Months (*N* = 45)		1 Year (*N* = 43)		2 Years (*N* = 35)		*p*-value
	N	%	N	%	N	%	N	%	N	%	
Medications for ED											
No	0	0.00	0	0.00	0	0.00	1	2.33	4	11.43	0.0027
Yes	48	100.00	46	100.00	45	100.00	42	97.67	31	88.57	
How often? (per week)	3.02 ± 0.62		2.71 ± 0.86		2.75 ± 1.25		2.34 ± 1.32		2.30 ± 1.07		0.0104
Any Vacuum Device											
No	44	91.67	13	28.26	13	28.89	17	39.53	21	60.00	<.0001
Yes	4	8.33	33	71.74	32	71.11	26	60.47	14	40.00	
How often? (per week)	4.50 ± 1.73		3.86 ± 1.97		3.01 ± 1.81		2.28 ± 1.67		3.12 ± 1.73		0.0137
Any Other Treatments (Injections)											
No	46	100.00	46	100.00	41	91.11	36	83.72	30	88.24	0.0072
Yes	0	0.00	0	0.00	4	8.89	7	16.28	4	11.76	
How often? (per week)					1.06 ± 0.83		0.92 ± 0.97		0.56 ± 0.52		0.6892

Barriers to adherence to the penile rehabilitation protocol were assessed. Identified factors from most to least common were as follows: cost (17.8–32.6%), inconvenience (2.1–31.1%), treatment is not very helpful (2.0–23.3%), lack of insurance coverage (6.7–22.5%), treatment is cumbersome (2.1–19.6%), timing of treatment is difficult (2.1–18.6%), side effects (4.2–11.1%). Barriers of cost and insurance coverage were unique to oral PDE5i therapy and the treatment being cumbersome was identified in men using a vacuum device. Other patient reported barriers to treatment included partner factors, frustration with lack of sexual function recovery, time constraints, traveling with therapy, comorbid health problems, delayed recovery, surgical complications and/or family stressors. High attrition rates for the PR program were noted with only 55.8% of men remaining at 12 months and 45.5% of men completing 24 months.

At the conclusion of the study, 88.6% of men still required medical treatment of their erectile dysfunction (*p* = 0.0027). Forty percent were using a VCD and 11.7% of men required intracavernosal injections, using them about once every 2 weeks.

## Discussion

Post-prostatectomy erectile dysfunction in a large population-based study revealed long term incidence of ED may be as high as 78–87% [3]. The role of oral PDE5i in penile rehabilitation has been called into question. The use of PR therapies has been found effective during utilization of the therapy, but it is uncertain if PR therapies can improve spontaneous erectile recovery [14]. Multiple variables may play a role in adherence to a PR therapy.

This observational cohort study followed a young population from a variety of prostate cancer surgeons from academic centers in the Chicago area for 2 years after radical prostatectomy. The majority of men underwent nerve sparing robotic assisted radical prostatectomy, which is consistent with current surgical trends [15, 16]. Continued improvement in erectile function was seen over the 24-month study period, as measured by SHIM, IIEF and erection hardness questionnaires. Erectile function is known to improve for 2 years post-operatively [17]. Pre-operatively, men were required to have a SHIM score of 16 to participate, and the average SHIM was 22.2 with 79% of men utilizing PDE5 inhibitors. Overall, the large majority of these men did not return to their baseline erectile function. Only 28.6% of men with normal erectile function pre-operatively (SHIM 22–25) returned to their baseline erectile function at 2 years. These results are similar to results from a recent study from Memorial Sloan Kettering, which determined erectile function returns in approximately 28% of post radical prostatectomy patients after 2 years [18]. The current study contributes further to the growing body of evidence that the majority of men do not recover their pre-operative erectile function and men undergoing nerve sparring radical prostatectomy should be counseled about this prior to treatment.

Penile stretched length was decreased by an average of 1.76 cm post-operatively and normalized after 6 months of penile rehabilitation which correlates with multiple studies [19, 20]. The men did regularly use PDE5i and the vacuum device if needed for penile rehabilitation and the improvement in penile length in the majority of patients could be due to this regimented rehabilitation program designed based on previous studies showing improvement [19, 21, 22]. Orgasm was described as absent or diminished in 65.1% of men post-operatively with return to normal or better in 62.9% of men at 24 months. These rates of anorgasmia and altered orgasmic sensation are consistent with available literature [23]. Men and their partners should be advised of these changes in orgasm prior to prostate cancer treatment.

Even amongst a highly educated population, adherence to therapy was less than expected. On average, men did not use the PDE5i at least 3 times a week, nor did they use the vacuum device daily. Barriers to adherence included cost of medications, side effects, remembering to utilize intermittent therapies, lack of perceived benefit, frustration with slow return of erectile function, inability to find time to use a cumbersome therapy, partner issues and life stressors. In general, the men in our program described many reasons why they were unable to consistently do penile rehabilitation as instructed. The more common reasons were consistent with previously reported barriers to therapy and adds further evidence about the challenges of doing penile rehabilitation and the reality of how much it can be done [24, 25]. Clinicians need to be realistic with men about what they can do with PR. It is important to identify barriers to treatment so they may be addressed in order to improve adherence. The most commonly identified barriers by men in our cohort were cost, perceived ineffectiveness of treatment and inconvenience.

Cost and insurance coverage were ongoing issues during the study period as the price of oral PDE5is continued to rise and Medicare no longer covered the vacuum device. Ultimately, men in the study were provided resources to obtain an affordable medical grade vacuum device and no participant in the study was without a vacuum device if they required one. Adherence to the penile rehabilitation protocol decreased with time for some men who reported improved erectile function, as expected. Conversely, some men identified frustration with lack of sexual function recovery as a barrier. Men who felt their progress was slow or inadequate were discouraged, reporting that rehabilitation treatments were a reminder of their erectile dysfunction, further deterring them from adhering to treatments. Inconvenience was a significant factor, with up to 31.1% of men stating that they were too busy for therapy. Additional inconveniences identified were related to travel and forgetfulness. One gentleman refused vacuum therapy due to his personal adversities. The challenges with adherence and the high attrition rate were also due to a number of reasons; need for adjuvant prostate cancer or other health issue treatments, partner factors, relationship issues, relocating away from the clinic, work environment and being too busy. At the conclusion of the study, nearly every participant continued to require therapy for ED. High attrition rates for the PR program were noted with only 44.5% completing the 24 month follow up.

Limitations of this study include the descriptive nature as well as selection bias. Only patients referred to our sexual health department were included. There was no control group in the study and this was not a randomized placebo trial, but rather a descriptive observational cohort study assessing sexual function before surgery, after surgery and with penile rehabilitation for 2 years. This is a major limitation of the study and randomized controlled studies are needed to assess the efficacy and safety of penile rehabilitation to be able to generalize findings to other populations. Following men for 2 years leads to attrition and the sample size was small and this was another limitation to the study.

Oral PDE5i medications are actually taken 2–3 time per week and VCD are often only used 2–4 time per week. Intracavernosal injections appeared to be used in an on-demand fashion, being utilized once every 1–2 weeks. This study describes how often patients are utilizing PR therapies. This information could be used in designing future studies and to help clinicians and patients be realistic about how often men are able to use oral PDE-5i, vacuum and injection therapy.

## Conclusion

On average erectile dysfunction was severe immediately following nerve sparring radical prostatectomy and improved over the 2 years. Most men did not return to baseline erectile function. This study contributes further to the growing body of evidence that the majority of men do not recover their pre-operative erectile function after nerve sparring radical prostatectomy even with penile rehabilitation. Orgasm also was diminished after surgery and improved over time. Despite comprehensive instructions and a frequent follow up schedule, PDE5i and VCD adherence was less than anticipated. The men identified many factors that impacted adherence to the PR program and/or attrition. Ultimately, men and their partners need to clearly understand the sexual ramifications of prostate cancer surgery on erectile function and orgasm. This information may help them to make informed decisions about penile rehabilitation and erectile dysfunction treatment options after surgery.

## Data Availability

The data generated from this study are not publicly available to protect the privacy of all participants. Further data output results may be provided only with permission and release from the authors.

## Appendix

### Instruments/Measures/Data Collection

**Side Effects:** Side effects were assessed through a checklist of side effects as identified in the prescribing information from the pharmaceutical company and the manufacturer of the vacuum device as well as a category of other where the participant can describe any other side effects from the medications or the vacuum device.

**Participant’s Perceived Barriers:** The participant were asked the question: What (if anything) about the medication and/or vacuum device you are using would inhibit or stop you from using them for treating your erectile dysfunction? The participant were provided space to respond with any perceived barriers to the treatment.

**Erection Hardness Grading Score (EHS):** Erection strength as rated on a scale ranging from 0 to 4 with 0 (representing a 0% erection) being no erection at all and 4 (representing a fully hard 100% erection) being a completely hard erection.

**The International Index of Erectile Function (IIEF) including the Sexual Health Inventory for Men (SHIM) and Overall Sexual Satisfaction:** The International Index of Erectile Dysfunction consists of 15-item questionnaire measuring five domains including erectile function, orgasmic function, sexual desire, intercourse satisfaction and overall sexual satisfaction, which were determined by principal components analysis. The Sexual Health Inventory for Men (SHIM) is a five item subscale within the International Index of Erectile Function pertaining to erectile dysfunction and was developed to determine the severity and presence of erectile dysfunction.

**The Self-Esteem and Relationship Questionnaire:** The Self-Esteem and Relationship Questionnaire is a 14-item instrument developed as a patient-reported tool to assess sexual confidence and intimacy in the sexual relationship.^7^

**Orgasm/climax:** In addition to questions used from the IIEF in the orgasm domain, orgasm/climax quality was evaluated. In addition to questions used from the IIEF in the orgasm domain, patients were asked to compare the quality of their orgasm to orgasms experienced prior to treatment. Response in terms of the quality of orgasm could be classified as better than before treatment, similar to before treatment or worst then before treatment.

**Other Documentation:** Demographics and Health History: Demographics and health history were obtained from the participant and/or the medical record. The prostate treatment was recorded. The participant’s health history in regards to factors that might affect erectile function was obtained from the participant or the medical record (i.e. cardiovascular disease, diabetes, neurological disorders, hyperlipidemia, medications that effect erectile function). The participant’s preoperative IIEF was obtained from the medical record or patient to assess erectile function prior to surgery. The patient reported use of any and all erectile dysfunction treatments in terms of how much, how often and what was used. The patients were asked to respond to an open ended question about any barriers to treatment. The patient were asked about ability to climax after prostate cancer treatment, urinary leakage during climax and the quality of their climax. The patient’s expectations and confidence about their erectile dysfunction treatment was assessed.

**Penile stretched length:** A ruler is pushed into the pubis directly above the penis and the stretched length was monitored for penile shrinkage during the clinical visit and that information as extracted from the patient visit.

## Abbreviations

ED: Erectile dysfunction
EDITS: Erectile Dysfunction Inventory of Treatment Satisfaction
EHS: Erection Hardness Score
ICI: Intracavernosal injections
IIEF: International Index of Erectile Function
PDE5i: Phosphodiesterase Type 5 inhibitor
PR: Penile Rehabilitation
QLI: Quality of Life Index Cancer Versioin
SD: Standard deviation
SHIM: Sexual Health Inventory for Men
VCD: Vacuum Constriction Device
